# Performance evaluation of three automated identification systems in detecting carbapenem-resistant *Enterobacteriaceae*

**DOI:** 10.1186/s12941-016-0154-0

**Published:** 2016-06-21

**Authors:** Qingwen He, Weiyuan Chen, Liya Huang, Qili Lin, Jingling Zhang, Rui Liu, Bin Li

**Affiliations:** Department of Clinical Laboratory, Fujian Medical University Union Hospital, 29 Xinquan Rd., Fuzhou, 350001 Fujian People’s Republic of China; Medical Technology and Engineering College, Fujian Medical University, Fuzhou, 350004 Fujian People’s Republic of China

**Keywords:** Carbapenem-resistant *Enterobacteriaceae*, Automated identification systems, PCR

## Abstract

**Background:**

Carbapenem-resistant *Enterobacteriaceae* (CRE) is prevalent around the world. Rapid and accurate detection of CRE is urgently needed to provide effective treatment. Automated identification systems have been widely used in clinical microbiology laboratories for rapid and high-efficient identification of pathogenic bacteria. However, critical evaluation and comparison are needed to determine the specificity and accuracy of different systems. The aim of this study was to evaluate the performance of three commonly used automated identification systems on the detection of CRE.

**Methods:**

A total of 81 non-repetitive clinical CRE isolates were collected from August 2011 to August 2012 in a Chinese university hospital, and all the isolates were confirmed to be resistant to carbapenems by the agar dilution method. The potential presence of carbapenemase genotypes of the 81 isolates was detected by PCR and sequencing. Using 81 clinical CRE isolates, we evaluated and compared the performance of three automated identification systems, MicroScan WalkAway 96 Plus, Phoenix 100, and Vitek 2 Compact, which are commonly used in China. To identify CRE, the comparator methodology was agar dilution method, while the PCR and sequencing was the comparator one to identify CPE.

**Results:**

PCR and sequencing analysis showed that 48 of the 81 CRE isolates carried carbapenemase genes, including 23 (28.4 %) IMP-4, 14 (17.3 %) IMP-8, 5 (6.2 %) NDM-1, and 8 (9.9 %) KPC-2. Notably, one *Klebsiella pneumoniae* isolate produced both IMP-4 and NDM-1. One *Klebsiellaoxytoca* isolate produced both KPC-2 and IMP-8. Of the 81 clinical CRE isolates, 56 (69.1 %), 33 (40.7 %) and 77 (95.1 %) were identified as CRE by MicroScan WalkAway 96 Plus, Phoenix 100, and Vitek 2 Compact, respectively. The sensitivities/specificities of MicroScan WalkAway, Phoenix 100 and Vitek 2 were 93.8/42.4 %, 54.2/66.7 %, and 75.0/36.4 %, respectively.

**Conclusions:**

The MicroScan WalkAway and Viteck2 systems are more reliable in clinical identification of CRE, whereas additional tests are required for the Pheonix 100 system. Our study provides a useful guideline for using automated identification systems for CRE identification.

## Background

Carbapenems are the most efficient antibiotics in treatment of serious infections caused by multidrug-resistant Gram-negative bacteria [1]. In recent years, the emergence and dissemination of carbapenem-resistant *Enterobacteriaceae* (CRE) have increased remarkably [2–5]. CRE are a subgroup of *Enterobacteriaceae* that are difficult to treat because of their high level resistance to antibiotics. *Klebsiella* species and *Escherichia coli* are common examples of *Enterobacteriaceae*, a part of the normal human gut bacteria that can become carbapenem-resistant. Resistance to carbapenems may be due to different mechanisms, and one of the mechanisms is through the production of carbapenemase which was called carbapenemase-producing Enterobacteriaceae (CPE) [6]. Carbapenemases are heterogeneous enzymes capable of hydrolysing cabapenems, such as imipenem and meropenem [6]. *Klebsiella pneumoniae* carbapenemase (KPC) were first described in *K. pneumoniae*, which were predominantly involved in *K. pneumoniae* and detected in other genera of the *Enterobacteriaceae* now [7–9]. In addition to KPC, other enzymes, such as NDM-1, VIM, and IMP, can also hydrolyze carbapenems and lead to the development of CRE [3, 8]. Further, CRE often has the characteristics of multi-drug resistant (MDR) and/or extensively drug-resistant (XDR), making it more difficult for effective treatment [9].

Therefore, rapid and accurate detection of carbapenem non-susceptible *Enterobacteriaceae* will play an important role in the infection control and prevention of multidrug resistance dissemination. The automated systems are widely used in many clinical laboratories for identification of bacterial species and antimicrobial susceptibility testing (AST). Phoenix 100, Vitek-2, and MicroScan WalkAway 96 Plus are the most common automated identification systems currently used in China [10–15]. However, critical evaluation of these systems is lacking to determine their sensitivity and specificity. Thus, the aim of the present study is to evaluate the abilities of the three systems to detect CRE.

## Methods

### Bacterial strains

A total of 81 non-repetitive clinical isolates of CRE were collected and confirmed from August 2011 to August 2012 at Fujian Medical University Union Hospital in Fuzhou, Fujian province, China. All the isolates had been preciously confirmed to be resistant to carbapenems (Imipenem, Meropenem and Doripenem) by the agar dilution method. These isolates included 18 *E. coli*, 36 *K. pneumoniae*, 21 *Enterobacter cloacae*, 1 *Enterobacter areogenes*, 4 *K. oxytoca*, and 1 *Serratia marcescens*.

### Detection of CRE and CPE by three automated systems

All the CRE isolates were tested duplicatelyin parallel on three automated identification systems. The three systems were: MicroScan WalkAway 96 Plus (SIEMENS AG FWB, Germany) with NC 50 cards for testing imipenem, meropenem and ertapenem; Phoenix 100 (Becton, Dickinson and Company, USA) with NMIC/ID-4 panels for testing imipenem and meropenem; and Vitek-2 Compact (Bio Mérieux, France) with AST-GN and AST-GN16 cards for testing imipenem and ertapenem simultaneously;. All the assays were accomplished in the Department of Clinical Laboratory, Fujian Medical University Union Hospital, Fuzhou, China. The carbapenemase-producing *Enterobacteriaceae* (CPE) were predicted by the advanced expert system (AES) of the automated systems. The susceptibility breakpoints of the three commercial systems were interpreted as recommended by the Clinical and Laboratory Standards Institute (CLSI) [16]. To identify CRE, the comparator methodology was agar dilution method, while the PCR and sequencing was the comparator one to identify CPE.

### DNA extraction

Genomic DNA of the CRE isolates was prepared by suspension of several colonies in 50 µl distilled water. The bacterial suspension was heated at 100 °C for 10 min to release the genomic DNA as described previously [17].

### PCR amplification and sequencing

The presence of carbapenemase genes was detected by PCR and sequencing. Gene analysis includes those encoding the class A carbapenemases (KPC, GES, SME, NMC-A and IMI), genes encoding metallo-β-lactamases (NDM-1, IMP, VIM, SPMand GIM), and genes encoding the OXA-type carbapenemase (OXA-48). The primers used in PCR were previously described [5]. All of the positive PCR products were sequenced and compared with the reported sequences in GenBank (http://www.ncbi.nlm.nih.gov/blast/).

## Results

The results of PCR and sequencing found that 48 of the 81 clinical CRE harbored carbapenemase genes, including IMP-4 (n = 23), IMP-8 (n = 14), NDM-1 (n = 5) and KPC-2 (n = 8). One *K. pneumoniae* isolate carries both IMP-4 and NDM-1 and one *K*. *oxytoca* isolate carries both KPC-2 and IMP-8. No carbapenemase genes of VIM-, NMC-, GIM-, SPM-, SME-, GES-, IMI- and OXA-48 were found in this study (Table 1). These genotypes were carried by different bacterial isolates, represented by 7 *E. coli* (38.9 %, 7/18), 26 *K. pneumoniae* (72.2 %, 26/36), 14 *E. cloacae* (66.7 %, 14/21), 3 *K. oxytoca* (75.0 %, 3/4) isolates. In addition, 1 *E. areogenes* isolate and 1 *S. marcescens* isolate has detected none carbapenemase genes (Table 1).

**Table 1:** Composition of the carbapenemase genotypes in the CRE isolates of this study

Species	IMP-4		IMP-8		NDM-1		KPC-2	
	Number	Percentage (%)	Number	Percentage (%)	Number	Percentage (%)	Number	Percentage (%)
*E. coli*	1	4.4	3	21.4	3	60.0	0	0.0
*K. pneumoniae* ^a^	13	56.5	5	35.7	2	40.0	6	75.0
*E. cloacae*	8	34.8	5	35.7	0	0.0	1	12.5
*E. areogenes*	0	0.0	0	0.0	0	0.0	0	0.0
*K. oxytoca* ^b^	1	4.4	1	7.1	0	0.0	1	12.5
*S. marcescens*	0	0.0	0	0.0	0	0.0	0	0.0
Total	23	100.0	14	100.0	5	100.0	8	100.0

^a^There was one *K. pneumoniae* isolate carrying both IMP-4 and NDM-1 enzymes

^b^There was one *K. oxytoca* carrying both KPC-2 and IMP-8 simultaneously

The three automated systems showed different ability of detecting/identifying CRE isolates in the study (Table 2). With AES, the MicroScan WalkAway and Vitek-2 showed a better sensitivity for the detection of CPE than the Phoenix 100. However, the Phoenix 100 AES had the best specificity for the detection of CPE (Table 3).

**Table 2:** Number of CRE isolates detected by each of the three automated identification systems in this study

Species	Number	Phoenix 100		Vitek-2 compact		WalkAway 96 plus	
		ID CRE	AES positive	ID CRE	AES positive	ID CRE	AES positive
*E. coli*	18	5	6	15	13	7	8
*K. pneumoniae*	36	16	19	36	34	31	33
*E. cloacae*	21	9	9	21	4	15	18
*E. areogenes*	1	0	0	1	1	0	1
*K. oxytoca*	4	3	3	4	4	3	4
*S. marcescens*	1	0	0	0	1	0	0
Total	81	33	37	77	57	56	64

Further, the three automated systems differed significantly in identification of the carbapenemase genotypes. The three automated systems showed high sensitivity in detection of NDM-1. All the isolates carrying NDM-1 were accurately identified. While the isolates with KPC-2 were all detected by the MicroScan WalkAway system, Two isolates were missed by the Vitek-2 and Phoenix systems, respectively. The (Table 3) one missed by Phoenix was susceptible to imipenem (MIC, ≤1 μg/ml) and meropenem (MIC, ≤1 μg/ml), while the other one missed by Vitek-2 was resistant to ertapenem (MIC, ≥8 μg/ml) and imipenem (MIC, 8 μg/ml). The three automated systems showed a relative low rate of identification for IMP. Out of the 37 IMP positive isolates, 21 were missed by the Phoenix, 11 were missed by the Vitek-2 Compact, and only three were missed in the MicroScan WalkAway system (Table 4).

**Table 3:** Performance of the three automated identification systems in detection of carbapenemase production in the CRE isolates of this study

Automated systems	AES positive/negative	No. of isolates with carbapenem resistance mechanism		Sensitivity (%)	Specificity (%)
		Carbapenemase (n = 48)	Noncarbapenemase (n = 33)		
Phoenix100	Positive	26	11	54.2	66.7
	Negative	22	22		
Vitek-2 compact	Positive	36	21	75.0	36.4
	Negative	12	12		
WalkAway 96 plus	Positive	45	19	93.8	42.4
	Negative	3	14

**Table 4:** Ability of the three automated systems in detection of the carbapenemase genotypes in the CRE isolates of this study

Carbapenemase genotypes	Number of isolates					
	Vitek-2 compact		MicroScan walkaway		Phoenix 100	
	Positive	Negative	Positive	Negative	Positive	Negative
IMP-4 (n = 23)	14	9	21	2	9	14
IMP-8 (n = 14)	12	2	13	1	7	7
KPC-2 (n = 8)	7	1	8	0	7	1
NDM-1 (n = 5)	5	0	5	0	5	0

## Discussion

CRE has been increasingly prevalent in China, and it is in critical need of rapid and accurate detection of CRE [18–20]. Automated identification systems have been demonstrated to have the ability to meet this requirement, but each system may perform differently, especially on identifying different genotypes of CRE [21–23].

In this study, we evaluated and compared the performance of three automated identification systems, MicroScan WalkAway, Phoenix 100 and Vitek-2, which are commonly used in China. The Vitek-2 system showed a higher identification rate than the MicroScan and the Phoenix on the identification of CRE. However, the MicroScan WalkAway’s AES showed a better sensitivity and specificity than the Vitek-2′s on the detection of CPE. Compared to the Vitek-2 and MicroScan WalkAway systems, the sensitivity of Phoenix 100 for the prediction of CRE and CPE needs to be improved.

The difference of accuracy in CRE detection among the three automated systems might be due to the number and type of carbapenems designed on the identification cards. The NC50 card of MicroScan WalkAway system has three carbapenems (imipenem, meropenem and ertapenem), the AST-GN card of Vitek-2 has two carbapenems (imipenem and ertapenem), and the NMIC/ID-4 card of Phoenix 100 also has two carbapenems (imipenem and meropenem). It was worth noting that Phoenix 100 incorporated with imipenem and meropenem had a higher specificity but a lower sensitivity compared to the other two systems. In a previous study, Woodford reported that the sensitivity/specificity of Vitek-2 N054 (containing ertapenem and meropenem) was 74/38 % [24], which is similar to the results in our study. We speculate that the combination of ertapenem with meropenem or imipenem has similar efficacy in the detection of CRE, but the absence of ertapenem has a decreased sensitivity. Also, different type of cards may carry different ability in CRE detection. Several studies have evalutated the different Vitek cards for detection of CRE, and found that there were different performance among those cards [25, 26].

In this study, four different carbapanemase genes (IMP-4, IMP-8, KPC-2 and NDM-1) had been detected. All of the three automated systems showed a high sensitivity in detecting KPC-2 and NDM-1, but low sensitivity to IMP, especially with the Phoenix 100 system. A previous study shows the Phoenix 100 system has a better performance in detecting IMP [24]. This difference may be due to the complex resistant phenotypes of isolates. In our study, the IMP producers missed by the Phoenix system were susceptible to imipenem and meropenem, and had lower MICs than those could be detected. Those IMP producers missed by Vitek-2 had higher MIC of ertapenem (≥8 μg/ml). Notably, there were 2 IMP producers missed by all of the three systems, and these isolates had lower MICs of imipenem, meropenem and ertapenem (≤1 μg/ml). Therefore, other methods should be performed together to increase the identification ability of IMP producers when these systems are using in clinical labs.

It should be noted that isolates determined as CRE by MicroScan WalkAway and Phoenix systems were less than those promoted as carbapenemase positive by AES. This result may be due to the sensitivity of the systems, which cannot discriminate some isolates with the intermediate of carbapanems. The shortcoming might be overcome via some complementary tests, such as addition of antimicrobial susceptibility discs and PCR.

Automated identification systems enable clinical microbiology laboratories to quickly and efficiently identify bacteria and characterize their drug susceptibilities. Automated identification systems should ideally achieve an accuracy rate of no less than 90 % in comparison to reference methods [27]. Our results suggest that the Vitek-2 and MicroScan WalkAway systems have better performance than the Pheonix 100 system on the identification of CRE, and the MicroScan WalkAway has the best sensitivity on the prediction of CPE. However, additional tests are required for the Pheonix 100 system.

In addition, our results indicate that the three systems carry different reliabilities depending upon the variety of the bacterial species. Previous studies suggest that both the Phoenix and the MicroScan WalkAway-96 systems are capable of providing accurate susceptibility test results for gram-negative bacilli with a wide array of antimicrobial agents [28]. However, our study showed that the Phoenix system was lower sensitivity in the identification of CRE than the others. Nonetheless, our findings suggest that while automated systems can provide rapid results for improved patient care, comparative studies should be conducted to evaluate the preference and reliability of the instruments for further improvement in their performance. Meanwhile, the results of our PCR screening showed limited diversity in the carbapenemase genes. However, they could covered the diversity of the kinds of resistance genes found in China [29, 30]. Therefore, our results will be of valuable reference in China.

## Conclusions

In conclusion, this is the first report that evaluates the performance of three automated identification systems commonly used in China. Our study will benefit clinical laboratories with the detection of CRE. And other tests should be combined to increase the accuracy when the three systems are used.

## Abbreviations

PCR: polymerase chain reaction
CRE: carbapenem-resistant *Enterobacteriaceae*
CPE: carbapenemase-producing *Enterobacteriaceae*
KPC: *Klebsiella pneumoniae* carbapenemase
NDM-1: New Delhi metallo-beta-lactamase-1
VIM: verona integron–encoded metallo-β-lactamase
IMP: active on imipenem
SPM: Sao Paulo metallo-β-lactamase
GIM: German imipenemase
GES: Guiana extended spectrum
SME: *Serratia marcescens* enzyme
NMC-A: not metalloenzyme carbapenemase
IMI: imipenem-hydrolyzingβ-lactamase
OXA-48: oxacillinase-48
MDR: multi-drug resistant
XDR: extensively drug-resistant
AST: antimicrobial susceptibility testing
CLSI: Clinical and Laboratory Standards Institute
AES: advanced expert system
MIC: minimum inhibitory concentration
